# Migratory marker expression in fibroblast foci of idiopathic pulmonary fibrosis

**DOI:** 10.1186/1465-9921-7-95

**Published:** 2006-06-30

**Authors:** Marco Chilosi, Alberto Zamò, Claudio Doglioni, Daniela Reghellin, Maurizio Lestani, Licia Montagna, Serena Pedron, Maria Grazia Ennas, Alessandra Cancellieri, Bruno Murer, Venerino Poletti

**Affiliations:** 1 Department of Pathology, University of Verona, Strada Le Grazie 8, 37134 Verona, Italy; 2 Department of Pathology San Raffaele Hospital, Via Olgettina60, 20132 Milan, Italy; 3 Department of Cytomorphology, University of Cagliari, Cittadella Universitaria 09042 Monserrato, Cagliari, Italy; 4 Department of Pathology, Maggiore Hospital, Largo B. Nigrisoli, 2, 40133 Bologna, Italy; 5 Department of Pathology, Umberto I Hospital, via Circonvallazione 50, 30173 Venice, Italy; 6 Department of Pneumology, G.B.Morgagni-L.Pierantoni Hospital, Via Carlo Forlanini 34, 47100 Forlì, Italy

## Abstract

**Background:**

Fibroblast foci (FF) are considered a relevant morphologic marker of idiopathic pulmonary fibrosis/usual interstitial pneumonia (IPF/UIP), and are recognised as sites where fibrotic responses are initiated and/or perpetuated in this severe disease. Despite their relevance, the cellular and molecular mechanisms responsible for the formation of FF and their role in tissue remodelling are poorly defined. In previous studies we have provided evidence of abnormal activation of the wnt-signaling-pathway in IPF/UIP that is centred on FF and the overlying epithelium. This important morphogenetic pathway is able to trigger epithelial-mesenchymal-transition (EMT), a mechanism involved in developmental and metastatic processes, which is also potentially involved in pulmonary fibrosis.

**Methods:**

Since EMT is characterised by enhancement of migratory potential of cells, we investigated the molecular profile of FF in 30 biopsies of IPF/UIP and a variety of control samples, focussing on the immunohistochemical expression of three molecules involved in cell motility and invasiveness, namely laminin-5-γ2-chain, fascin, and heat-shock-protein-27.

**Results:**

We provide evidence that in UIP these three molecules are abnormally expressed in discrete clusters of bronchiolar basal cells precisely localised in FF. These cellular clusters expressed laminin-5-γ2-chain and heat-shock-protein-27 at very high levels, forming characteristic three-layered lesions defined as "sandwich-foci" (SW-FF). Upon quantitative analysis SW-FF were present in 28/30 UIP samples, representing more than 50% of recognisable FF in 21/30, but were exceedingly rare in a wide variety of lung pathologies examined as controls. In UIP, SW-FF were often observed in areas of microscopic honeycombing, and were also found at the interface between normal lung tissue and areas of dense scarring.

**Conclusion:**

These molecular abnormalities strongly suggest that SW-FF represent the leading edge of pulmonary remodelling, where abnormal migration and re-epithelialisation take place, and that abnormal proliferation and migration of bronchiolar basal cells have a major role in the remodelling process characterising IPF/UIP. Further investigations will assess their possible use as reliable markers for better defining the UIP-pattern in difficult cases.

## Background

Idiopathic pulmonary fibrosis (IPF) is the most common and severe idiopathic interstitial pneumonia [1,2]. In affected portions of the lung irreversible remodelling of tissue architecture takes place, which is histopathologically described as "*usual interstitial pneumonia*". In recent years evolving opinions regarding the pathogenesis of this specific chronic fibrosing disease have arose and different models have been proposed [3-6]. Recently, the "inflammatory theory" of IPF/UIP has been challenged on the assumption that abnormal epithelial-mesenchymal interactions and aberrant wound healing are in fact crucial pathogenetic events [3,4]. Although this new scheme is appealing, many points remain unresolved, including the nature of "fibroblast-foci" (FF), as well as the molecular mechanisms responsible for alveolar loss, honeycomb modifications, abnormal fibrosis and severe tissue remodelling. In previous studies we provided evidence that the wnt/β-catenin signalling pathway is abnormally activated in IPF/UIP, acting on both the alveolar and bronchiolar components [7-9]. The central role played by the wnt/β-catenin pathway in lung embryogenesis and pathology is further demonstrated by the complex functions exerted by this pathway in regulating a variety of crucial mechanisms, including cell proliferation, apoptosis, cell migration, and angiogenesis [10]. Accordingly, the wnt-signalling pathway regulates branching morphogenesis in the lung, and can produce severe modifications in the developmental potential of embryonic lung differentiation when aberrantly expressed [11]. Interestingly, the wnt/β-catenin pathway is a central trigger of epithelial-mesenchymal transition (EMT), an important process occurring during critical phases of embryonic development, tumour progression, and fibrotic tissue repair in different organs including the lung [12-14]. This possibility is relevant since such a new scheme could completely change the pathogenic scenario for IPF/UIP, a devastating disease where new therapeutic options are necessary [15].

We hypothesise that uncontrolled activation of wnt-β-catenin pathway can profoundly influence tissue remodelling in IPF/UIP by triggering pronounced cell migration and proliferation at sites of aberrant expression, thus interfering with the physiologic molecular program determining correct tissue repair. A variety of molecules involved in cell migration and invasion are in fact targets of β-catenin transcriptional activation and/or regulation, including matrylisin/MMP7 (a metalloproteinase with multifunctional roles including the induction of epithelial cell migration, apoptosis and metaplastic conversion) [9,16,17], laminin-5 gamma-2 chain (LAM5γ2; a potent migration-inducing factor expressed by epithelial cells in healing tissues) [18], tenascin-C (a component of the extracellular matrix expressed during development, neoplastic invasion and wound-healing) [19], and fascin (an actin-binding protein involved in cell motility of epithelial cells) [20,21]. Abnormal expression of matrilysin/MMP7 has been demonstrated in UIP samples by both analysis of gene expression and immunohistochemistry [9,22], and tenascin has been found to be expressed at high levels in fibroblast foci. However, only limited information is available in UIP regarding the expression of other molecules involved in cell migration, such as LAM5γ2, fascin, and heat-shock protein-27 (HSP27), a multifunctional stress-inducible molecule involved in the modulation of actin microfilament dynamics and cell migration [23-25].

In this study we have investigated the immunohistochemical expression of LAM5γ2, fascin, and HSP27 in 30 cases of UIP, and in a large variety of biopsies of other pulmonary diseases used as controls.

## Methods

All studies were carried out in compliance to the Helsinki declaration and in accordance with Italian law, following the ethical recommendations of the Institutions where they were performed.

### Study population

The study group consisted of 30 previously untreated patients with clinical, radiographic, physiologic and bronchoalveolar-lavage findings consistent with a diagnosis of IPF. Histological examination of surgical lung biopsies revealed all the major features of UIP according to recently defined criteria [1,2]. Controls included normal lung tissue (n = 5), and a variety of pathologic samples retrieved from our files. Among these, a series of biopsies were investigated showing UIP-like modifications: allergic extrinsic alveolitis (n = 2), autoimmunity (n = 3), and amiodarone toxicity (n = 1) also in addition to samples showing extensive scarring with fibroblast foci (recurrent pneumothorax, n = 5; carcinoma, n = 5; post-infection, n = 2). Diffuse parenchymal lung diseases were also investigated as controls, including non-specific interstitial pneumonia (NSIP, n = 5), cryptogenic organizing pneumonia (COP, n = 10), acute interstitial pneumonia with diffuse alveolar damage (AIP/DAD, n = 3), desquamative interstitial pneumonia (DIP, n = 4), extrinsic allergic alveolitis (EAA, n = 8), Langerhans' cell histiocytosis (LCH, n = 3), acute eosinophilic pneumonia (AEP, n = 2), and airway-centred interstitial fibrosis (ACIF, n = 3). All these cases were defined according to the most recent diagnostic criteria [1,2]. Diseases where severe airway remodelling is a common feature were also included as controls (diffuse panbronchiolitis, DPB, n = 1; constrictive bronchiolitis, n = 2).

### Immunohistochemical staining and antibodies

Serial sections of UIP and control cases were immunostained with monoclonal antibodies recognizing laminin-5 gamma-2 chain (clone-4G1, DakoCytomation, Glostrup, Denmark), fascin (clone-55K-2, DakoCytomation), and two different antibodies recognising heat-shock-protein-27 (clone-2B4, Novocastra, Newcastle, UK) and its phosphorylated form (S82, a rabbit monoclonal antibody recognising HSP27 phosphorylated on serine-82, Epitomics-Inc, Burlingame, CA). Heat-induced antigen retrieval was performed for 4G1, 55K-2 and S82 antibodies using a microwave oven and citrate buffer 0.01 M pH7.0 (4G1), and pH6.0 (55K-2 and S82) respectively, whereas no treatment was used for 2B4 antibody. All samples were processed using a sensitive avidin-streptavidin-peroxidase technique (Biogenex San Ramon, CA) in an automated staining system (GenoMx i6000, BioGenex). Sections incubated without the primary antibody served as a negative control.

To better define the nature and level of differentiation of the epithelial and mesenchymal lesions, we utilized antibodies recognizing cytokeratin-8/18 as a pan-epithelial marker, cytokeratin-5 (CK5) as a basal-cell marker, ΔN-p63 isoform for bronchiolar basal cells, tenascin and α-smooth-muscle actin (SMA) for fibroblast foci, CC10 for clara-cells, and surfactant apoprotein-A (SPA) for pneumocytes (see Table 1 for details). Double-marker analysis was also performed in selected samples using either anti-tenascin or anti-ΔN-p63 antibodies, combined with 4G1 or 2B4 antibodies, in order to define the precise location of LAM5γ2 and HSP27 immunoreactivity. These double-marker methods have been previously described in detail [7,26]. A similar technique was used for demonstrating LAM5γ2 and tenascin.

**Table 1:** Antibodies utilised in this study

***Antigen***	***Clone/Ab***	***Reactivity***	***Source***
heat shock protein 27	clone 2B4	FF* in UIP	Novocastra
Heat shock protein 27, phosphorylated on serine-82	S82, rabbit monoclonal	FF in UIP	Epitomics-Inc,
laminin-5γ-2 chain	4G1	FF in UIP	DakoCytomation
fascin	55K-2	FF in UIP, vessels, myofibroblasts, dendritic cells	DakoCytomation
α-smooth muscle actin	1A4	smooth muscle, myofibroblasts in FF	DakoCytomation
CC10	urine protein 1 (rabbit polyclonal)	Clara cells	DakoCytomation
cytokeratin 8/18	clone 5D3	epithelial cells	Biogenex
cytokeratin 5	clone XM26	bronchiolar basal cells	Novocastra
ΔN-p63 truncated isoform	p40 (rabbit polyclonal)	bronchiolar basal cells	Oncogene Research
surfactant-apoprotein A (SP-A)	clone PE-10	pneumocytes	DakoCytomation
tenascin	TN2	FF in UIP	DakoCytomation

*FF: fibroblast foci

## Results

All the 30 samples of IPF were morphologically described as usual interstitial pneumonia by the presence of typical modifications. These included patchy interstitial fibrosis alternating with normal or minimally affected parenchymal tissue, and honeycombing. FF, morphologically defined as circumscribed collections of loose organizing connective tissue formed by spindle-shaped myofibroblasts, was present in all samples, and quantified on serial sections by immunostaining for αSMA and tenascin. The number of FF was highly variable in different samples, ranging from 1 to >10 per cm^2^. The epithelial cells overlying FF were heterogeneous and appeared as either flat, cuboidal, or ciliated.

### Laminin-5 gamma-2 chain expression pattern in IPF/UIP samples and controls

**Figure 1:**
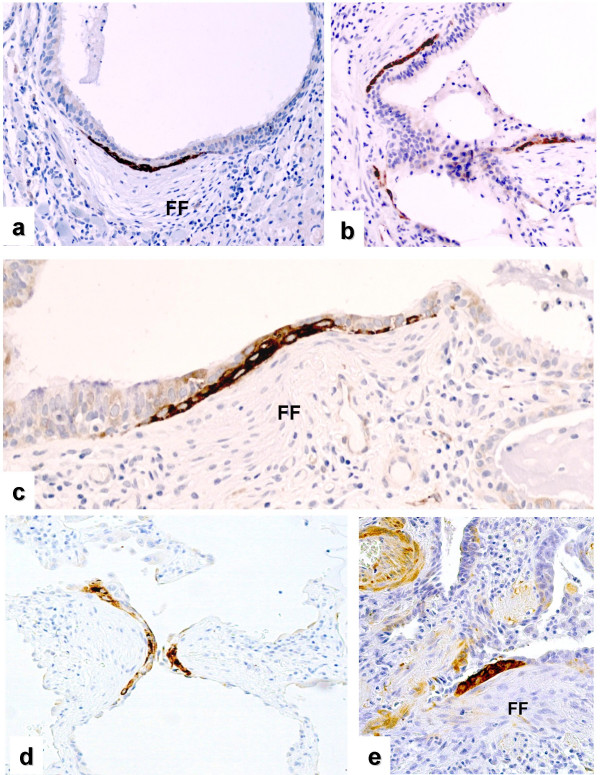
**LAM5γ2 and HSP27 expression in FF of UIP biopsies**. Expression of LAM5γ2 (**a**, **b**, and **c**) and HSP27 (**d**, and **e**) in different cases of IPF/UIP. The immunoreactivity is similar for the two molecules, mainly restricted to basal cell sheets located between luminal bronchiolar cells and myofibroblast clusters of fibroblast foci (*sandwich*-FF).

**Figure 2:**
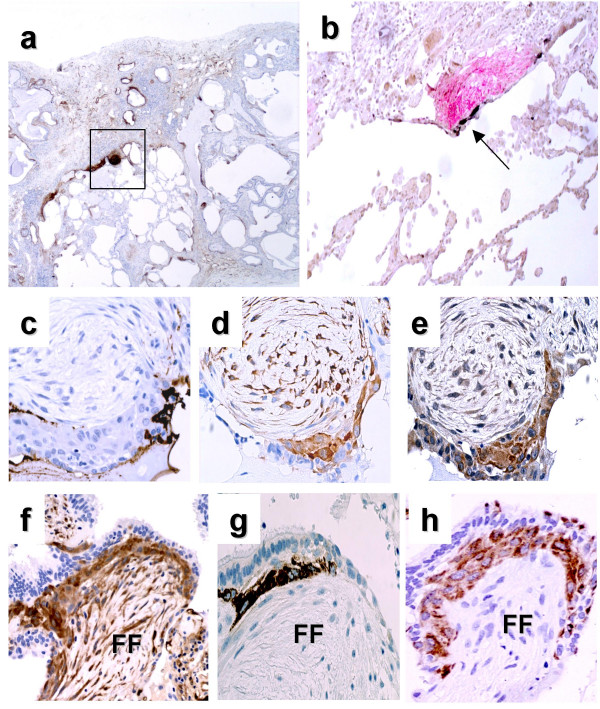
**Characterisation on serial sections of the cells expressing LAM5γ2, fascin and HSP27 in "*sandwich*-FF" of UIP biopsies**. (**a**) A fibroblast focus is shown by tenascin immunostaining in a UIP biopsy at the edge between dense scarring and scarcely involved lung (square frame). In (**b**) The same FF is analysed for HSP27 (brown) and tenascin (red) by double-marker immunostaining. The same lesion was studied using serial sections, showing that surfactant SPA is not expressed by overlying epithelial cells (**c**), but a cluster of basal cells expresses high level of fascin (**d**) and MMP7/matrylisin (**e**). In **d**myofibroblasts show discrete immunoreactivity for fascin. In the **f-g-h**sequence a "*sandwich-*FF" is shown, immunostained on serial sections for fascin (**f**), LAM5γ2 (**g**), and keratin-5, a marker of bronchiolar basal cells (**h**).

**Figure 3:**
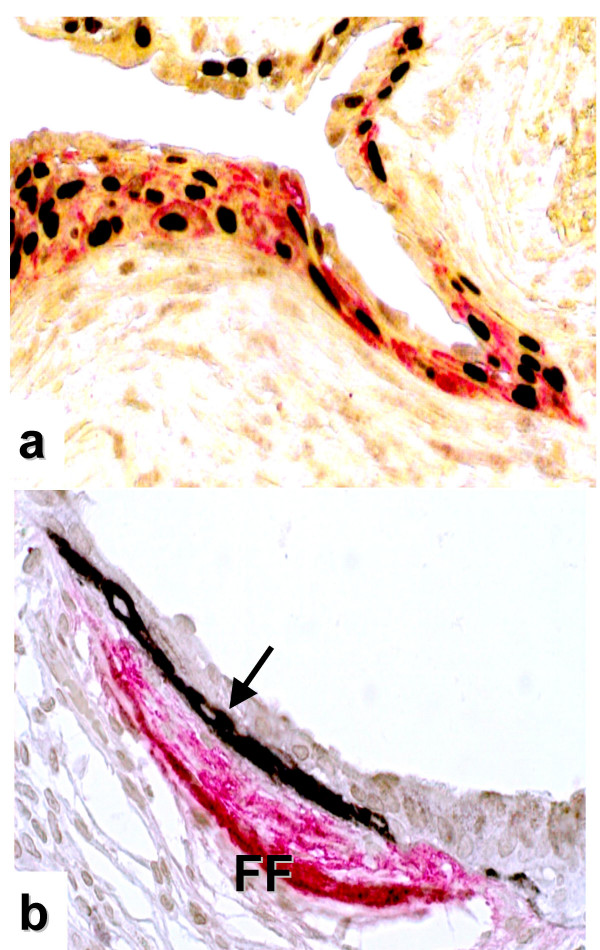
**Characterisation of "*sandwich*-FF" by double-marker immunostaining in UIP biopsies**. In (**a**) nuclear immunoreactivity of ΔN-p63 (brown-black), a well established marker of bronchiolar basal cells, clearly defines the nature of the cells expressing LAM5γ2 (cytoplasmic red immunoreactivity). In (**b**), another sandwich lesion immunostained by the double marker technique and showing strong expression of tenascin in the cluster of myofibroblasts is seen (red). The cluster of basal cells located between tenascin+ myofibroblasts and negative luminal bronchiolar cells strongly expresses LAM5γ2 (arrow).

A definite cell population exhibiting strong LAM5γ2 immunoreactivity was observed in IPF/UIP samples, formed by positive cells localized within structures recognised as fibroblast foci by morphology and tenascin expression (Figs. 1, 2, 3). These cells had the immunophenotypic characteristics of bronchiolar basal cells (SPA-, CK5+ and ΔNp63+ on serial sections and double-immunostaining)(Figs. 2 and 3), formed linear sheets or small aggregates of LAM5γ2+ cells overlying FF, and were characteristically located between negative luminal sheets of bronchiolar epithelial cells and negative myofibroblasts (Figs. 1, 2, 3). Due to this characteristic pattern, these FF were termed "sandwich-FF" (SW-FF). SW-FF were demonstrated in 28/30 of UIP samples and the two negative cases contained only rare FF. Quantitative analysis of SW-FF showed that they were dependent on the area of tissue available and the number of FF (range 0.5 – 10 × cm^2 ^of sample tissue). SW-FF accounted for more than 50% of FF in 21/30 cases, were less than 50% of FF in 7/30 samples, and were totally absent in the remaining 2/30 cases. The epithelial cells overlying the non SW-FF exhibited the phenotype of alveolar pneumocytes (SPA+, CK5-). SW-FF were also present in 5 end-stage cases where dense scarring and honeycombing were prevalent, located within the wall of microscopic honeycomb cysts. In all UIP samples LAM5γ2 expression was not observed in basal cells of normal bronchioles, or in bronchioles exhibiting proliferative modifications (bronchiolisation, basal cell hyperplasia, squamous metaplasia).

Focal LAM5γ2 cytoplasmic expression was observed in scattered atypically enlarged type-II pneumocytes at sites of tissue damage in all cases of IPF/UIP.

#### Control samples

**Figure 4:**
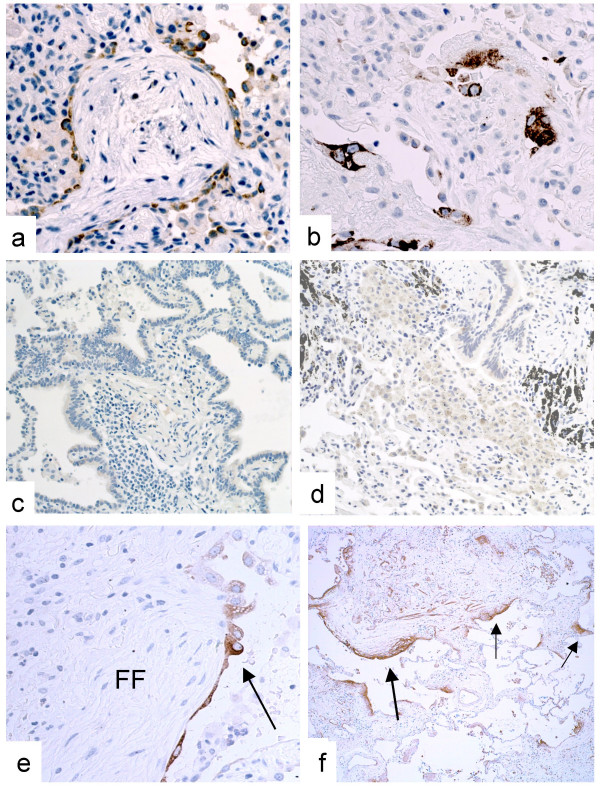
**LAM5γ2 expression in control samples**. LAM5γ2 expression is observed in a subset of regenerating epithelial cells in cryptogenic organising pneumonia (COP, **a**), and diffuse alveolar damage/acute interstitial pneumonia (AIP/DAD, **b**), but is completely absent in allergic extrinsic alveolitis (AEA, **c**) and desquamative interstitial pneumonia (DIP, **d**), used as controls. In this UIP-like case (systemic sclerosis LAM5γ2 immunoreactivity was restricted to pneumocytes overlying FF (**e**, arrow), but the "sandwich" pattern was not observed despite the high number of fibroblast foci, as shown by tenascin immunostaining on serial sections (**f**, arrows).

LAM5γ2 expression was absent in all samples of normal lung and was carefully evaluated in both the bronchiolar and alveolar components in a variety of pulmonary disorders. In particular, we focused on diseases characterised by centrolobular involvement (hypersensitivity pneumonitis, Langerhans' cell histiocytosis, air-centred interstitial fibrosis/ACIF), by the occurrence of extensive bronchiolar remodelling (diffuse panbronchiolitis and constrictive bronchiolitis), or by the presence of epithelial damage and organising connective tissue (AIP/DAD, AEP, OP/COP, NSIP, autoimmune lung diseases, scarring lesions). None of the bronchiolar cells in any of these pulmonary biopsies expressed LAM5γ2 (Fig. 4), with the exception of two cases characterised by extensive scarring (one pulmonary carcinoma and one recurrent pneumothorax), where scattered lesions resembling SW-FF could be focally observed in enlarged bronchiolar structures.

In control cases focal LAM5γ2 immunoreactivity was observed in abnormal/hypertrophic pneumocytes at sites of alveolar damage (Fig. 4). Epithelial cells covering intra-alveolar fibroblastic polyps (Masson's bodies) in OP/COP samples variably expressed LAM5γ2 (Fig. 4), never exhibited the sandwich pattern observed in UIP, and were characterised by an alveolar pneumocyte immunophenotype (SPA+, CK5-negative, ΔN-p63-negative).

### HSP27 and phospho-HSP27 expression in IPF/UIP and controls

The immunoreactivity pattern observed with HSP27 and phospho-HSP27 antibodies was practically identical to that observed for LAM5γ2 expression (Figs. 1, 2, 3). Accordingly, HSP27 was absent in normal control lung, with focal expression at sites of pneumocyte regeneration in pathologic samples. IPF/UIP foci exhibiting the sandwich pattern were demonstrated at the same frequency and location as observed with LAM5γ2 on serial sections, using both 2B4 and S82 antibodies.

### Fascin expression pattern in IPF/UIP samples and controls

The epithelial basal cells overlying FF exhibited elevated levels of fascin, with a distribution similar to that observed with LAM5γ2 and HSP27 (Fig. 2). These fascin expressing cells were clearly recognised as CK5+, ΔN-p63+ basal cells upon immunophenotypical analysis of serial sections (Fig. 2). Fascin expression was more widespread than LAM5γ2 and HSP27, and different mesenchymal cell components expressed this protein, including blood vessels and myofibroblasts (Fig. 2). For this reason the sandwich pattern could not be easily recognised using fascin immunostaining. All types of epithelial cells including bronchiolar and alveolar cells were negative for fascin in control samples, with the exception of regenerating pneumocytes in pulmonary diseases where alveolar damage was observed (AIP/DAD, COP, EAA, etc.).

### Co-expression of wnt-β-catenin target gene products

When analysed on serial sections, basal cells expressing the three molecules (LAM5γ2, HSP27 and fascin) in fibroblast foci of UIP samples also expressed nuclear β-catenin and matrilysin, as previously described [9] (Fig. 2).

## Discussion

The pathogenesis of idiopathic interstitial pneumonia is poorly defined and remains the subject of intense debate and research. Although high-throughput molecular analysis has been applied to UIP samples with some success [22,27], the precise definition of molecular events occurring at sites of disease activity will require direct *in situ *analysis of lung biopsies.

In this paper we provide *in situ *evidence that the epithelial component overlying fibroblast foci (FF) expresses a set of molecules involved in inducing cell motility and invasiveness, including LAM5γ2, fascin and HSP27. The relevance of our findings is related to the specific functions of the investigated molecules, as well as the characteristic tissue localisation of their abnormal expression. The morphology and location of this cellular component is in fact particularly intriguing, since positive cells appeared as linear clusters of bronchiolar basal cells within FF, wedged between luminal epithelial cells and myofibroblasts. The recognition of these *negative-positive-negative *three-layered lesions (that we termed "*sandwich" fibroblast-foci *or SW-FF) was particularly evident using LAM5γ2 and HSP27 as markers (Figs. 1 and 2).

The trimeric protein laminin-5 (α3, β3, γ2-chain) is an integral part of the basal lamina of stratified epithelia where it plays a crucial role in the organization of the basal stem-cell niche by providing epithelial-mesenchymal connections by interacting with integrin α6β4 [28]. These interactions are critical for regulating cell migration, an event required in different processes, such as wound healing, embryogenesis and metastatic dissemination [29]. The γ2 chain of laminin-5 (LAM5γ2) acts as a soluble cell motility factor in a variety of conditions after its cleavage by metalloproteinases, and enhanced expression of LAM5γ2 is considered one of the best marker of invasiveness in different carcinomas [30-33]. At the invasive front of colorectal carcinoma the cytoplasmic accumulation of LAM5γ2 in neoplastic cells is the result of synergistic activation of the *LAMC2 *gene by β-catenin, TGFβ1, and hepatocyte-growth factor (HGF), molecules that all have been variably involved in the pathogenesis of IPF/UIP [9,18,34-36]. Our findings regarding LAM5γ2 expression are partially at variance with those recently described by Lappi-Bianco et al [37], who observed LAM5γ2 expression in regenerating epithelial cells in both COP and IPF/UIP, but did not note the characteristic immunoreactivity in basal cells at FF. In our study, the bronchiolar nature of epithelial cells overlying FF was assessed by sensitive and specific immunophenotyping using recently-introduced robust markers such as ΔN-p63, that were not used in the aforementioned study, thus possibly explaining this apparent discrepancy.

Fascin is a 55 kD protein that binds actin, organising it into well ordered bundles thus contributing to the formation of the various cell protrusions (filopodia, spikes, lamellipodial ribs and dendrites) necessary for cell adhesion and motility [21,38]. Fascin is not normally expressed in pulmonary epithelial cells, but is up-regulated in a number of carcinomas [39,40]. Interestingly, fascin can also associate with β-catenin, utilising the same binding sites used by E-cadherin and co-localising at cell-cell borders and leading edges [20].

Heat shock protein-27 is a small molecule rapidly induced and phosphorylated by heat shock and other stressing agents [41]. HSP27 behaves as an actin-capping protein interfering with its polymerisation, thus regulating cell adhesion and motility under the control of p38 MAPK (p38 mitogen-activated protein kinase) [24,25,42]. In addition, HSP27 can mediate resistance against cell death induced by stress and differentiation [43,44]. The mechanisms accounting for the cytoprotective functions of HSP27 are complex, since HSP27 directly interacts with several apoptotic effectors. Using a specific antibody, we demonstrated that the HSP27 protein expressed at FF is phosphorylated, arguing in favour of its biological functionality.

Recent pathogenic models of IPF/UIP have been proposed, suggesting that disturbed re-epithelialisation occurs at sites of abnormal tissue damage and repair [3,4,7]. The demonstration of increased cell migration at sites of ongoing remodelling is in line with these models, and also with the abnormal wnt-pathway activation occurring at the same sites as previously suggested by us (9).

Finally, according to our data, the sandwich-foci observed in the large majority of UIP samples using LAM5γ2 and HSP27 antibodies could represent a useful new marker for characterisation of IPF/UIP. The UIP pattern, although well defined in its morphological features, is not completely specific for IPF, and both mimicking and difficult cases arise. Accordingly, full diagnostic agreement regarding IPF/UIP evaluation on lung biopsy is not reached even among expert lung pathologists [45]. The sandwich-pattern is easily recognisable on routine tissue samples and high quality antibodies for both LAM5γ2 and HSP27 are available. Further studies are in progress to validate the utility of this promising marker in the differential diagnosis of interstitial pneumonias on a larger series of cases.

## Conclusion

The molecular abnormalities demonstrated in this study suggest that abnormal proliferation and migration of epithelial basal cells overlying myofibroblasts in FF have a major role in the pathological remodelling characterising IPF/UIP, leading to bronchiolar colonisation with substitution of the alveolated parenchyma and eventual progression towards lung fibrosis and functional loss. Activation of the wnt-pathway and increased expression of proteins involved in cell migration and invasiveness are involved in this process.

## Abbreviations

FF: fibroblast foci

IPF/UIP: idiopathic pulmonary fibrosis/usual interstitial pneumonia

EMT: epithelial-mesenchymal transition

SW-FF: sandwich-fibroblast foci

UIP: usual interstitial pneumonia

IPF: idiopathic pulmonary fibrosis

MMP7: matrix metalloproteinase 7

LAM5γ2: laminin-5 gamma-2 chain

HSP27: heath-shock protein 27

NSIP: non-specific interstitial pneumonia

AIP/DAD: acute interstitial pneumonia with diffuse alveolar damage

DIP: desquamative interstitial pneumonia

EAA: extrinsic allergic alveolitis

LCH: Langerhans cell histiocytosis

AEP: acute eosinophilic pneumonia

ACIF: airway-centred interstitial fibrosis

DPB: diffuse panbronchiolitis

CK5: cytokeratin 5

SMA: smooth muscle actin

SPA: surfactant apoprotein A

OP/COP: organising pneumonia/cryptogenic organising pneumonia

MAPK: mitogen activated protein kinase

## Competing interests

The author(s) declare that they have no competing interests.

## Authors' contributions

MC designed the study, evaluated slides microscopically, drafted and edited the manuscript. AZ participated in study design, manuscript drafting and revision. DR collected study specimens and helped in manuscript revision. ML evaluated slides microscopically and revised the manuscript. LM and SP carried out the immunoassays. MGE evaluated slides microscopically and revised the manuscript. AC selected patients for inclusion in the study and revised the manuscript. BM evaluated slides microscopically and revised the manuscript. VP participated in study design, selected patients for inclusion in the study and revised the manuscript.
